# Stem Cell Applications and Tenogenic Differentiation Strategies for Tendon Repair

**DOI:** 10.1155/2023/3656498

**Published:** 2023-03-15

**Authors:** Ziyang Yuan, Haomiao Yu, Huibin Long, Yike Dai, Lin Shi, Jiaming Zhao, Ai Guo, Naicheng Diao, Lifeng Ma, Heyong Yin

**Affiliations:** Department of Orthopaedics, Beijing Friendship Hospital, Capital Medical University, Beijing 100053, China

## Abstract

Tendons are associated with a high injury risk because of their overuse and age-related tissue degeneration. Thus, tendon injuries pose great clinical and economic challenges to the society. Unfortunately, the natural healing capacity of tendons is far from perfect, and they respond poorly to conventional treatments when injured. Consequently, tendons require a long period of healing and recovery, and the initial strength and function of a repaired tendon cannot be completely restored as it is prone to a high rate of rerupture. Nowadays, the application of various stem cell sources, including mesenchymal stem cells (MSCs) and embryonic stem cells (ESCs), for tendon repair has shown great potential, because these cells can differentiate into a tendon lineage and promote functional tendon repair. However, the mechanism underlying tenogenic differentiation remains unclear. Moreover, no widely adopted protocol has been established for effective and reproducible tenogenic differentiation because of the lack of definitive biomarkers for identifying the tendon differentiation cascades. This work is aimed at reviewing the literature over the past decade and providing an overview of background information on the clinical relevance of tendons and the urgent need to improve tendon repair; the advantages and disadvantages of different stem cell types used for boosting tendon repair; and the unique advantages of reported strategies for tenogenic differentiation, including growth factors, gene modification, biomaterials, and mechanical stimulation.

## 1. Introduction

Tendons are integral to the musculoskeletal system, connecting and transmitting force from muscles to bones. Because of their unique composition and structure, tendons can store elastic energy and endure high tensile forces, which make movement possible [1]. Along with aging, the function of tendons declines, and they become more susceptible to degenerative diseases and injuries [2]. Due to the aging population and growing participation of people in sports, tendon injuries are becoming common and are posing clinical and economic challenges to society [3]. These injuries are frequently a result of trauma, chronic overuse, and age-related tissue degeneration [4] and cause a series of physical and social problems, such as pain, disability, increased medical expenses, and decreased productivity [5]. Unfortunately, these injuries respond poorly to conventional treatments, such as medication, physical therapy, and suturing. Stem cell-based therapies have exhibited considerable potential for boosting tendon healing. However, stem cell application and its clinical translation are hampered owing to the lack of a widely adopted protocol for effective and reproducible tenogenic differentiation. Unlike bone, cartilage, and muscle, tendon is among the least understood musculoskeletal tissues in terms of developmental biology and the tissue healing process [6]. Although several tendon-specific biomarkers, such as scleraxis (Scx) and mohawk (Mkx), have been identified, the ontogeny of the tenogenic lineage and signaling cascades in tendon differentiation need further clarification [7]. Identifying additional master tendon transcription factors will be of great significance for understanding tendon biology and tenogenic differentiation. The main work of this review is to provide background information on the clinical relevance of tendons, accentuate the limited self-healing capacity of the tendon tissue, illustrate the urgent need to improve tendon repair, introduce different stem cell types used for boosting tendon repair, and summarize various methods reported for tenogenic differentiation.

## 2. Stem Cell Sources for Tendon Repair

### 2.1. Tendon Clinical Relevance

**Figure 1:**
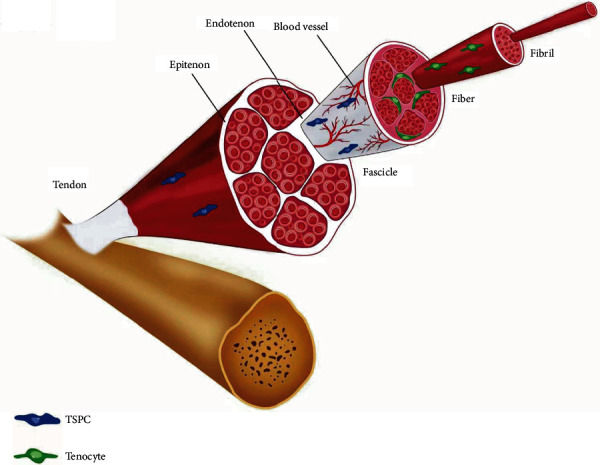
Schematic presentation of basic tendon structure. Plenty of microfibrils aggregate into fibrils that progressively group together into fibers arranging from head to tail in the tendon. Bundles of fibers are ensheathed by endotenon, which enables fiber groups to slide against each other with low friction and also transports blood vessels for nourishing the deeper part of the tissue. Fiber bundles then group together into fascicles and encompassed by endotenon. Finally, fascicles are bound together by the epitenon to develop the tendon.

Tendon is a dense connective tissue highly organized in a hierarchical manner. It is mainly composed of collagen fibers, elastin, and tendon cells embedded in the proteoglycan–water matrix [8] (Figure 1). Tendinopathy is a clinical condition widely distributed around the globe. This condition is related to tendon degeneration or failure of healing after injuries due to continuous overload [9, 10]. Certain tendons, such as the rotator cuff, Achilles tendon, and patellar tendon, have a higher tendency to cause pathological damage based on degenerative processes and overuse [10]. Achilles tendinopathy affected 1.85 in 1,000 Dutch general practitioner-registered patients, and the risk of Achilles tendon injury in adults (21–60 years) is reported to be as high as 2.35 in 1,000 [11]. Moreover, tendon injuries pose a substantial economic burden to individuals and society. In the United States, the cost of tendon injury treatment reaches 30 billion U.S. dollars annually, and in Europe, it reaches more than 115 billion Euros per year [12].

Given the low cell density, low vascularity, and low metabolic activity of tendon tissue, tendon repair after an injury is extraordinarily ineffective and inefficient [13, 14]. Moreover, a repaired tissue in most patients, particularly those in elderly patients, typically fails to restore its initial structure and mechanical strength. Consequently, the quality and function of repaired tendons are inferior to those of healthy tendons [10]. At present, the most frequently used strategies for repairing injured tendons are conservative treatments, such as immobilization and medication, as well as surgical intervention for full tendon rupture, such as suturing. However, these treatment methods are associated with unsatisfactory long-term clinical outcomes, with ectopic calcification, restrictive adhesions, and high rerupture occurrence [15]. Hence, novel treatments, such as stem cell-based therapies for boosting tendon healing, have recently attracted substantial attention.

Stem cells can renew themselves and can differentiate into specific cell lineages when induced appropriately [16]. These stem cells are also highly proliferative and exhibit high biosynthesis activities. They have the potential to differentiate into tenocytes. Moreover, they secrete paracrine factors and exert immunomodulatory effects for boosting tendon healing. Thus, stem cell application for tendon repair is considered a promising strategy [17].

### 2.2. Stem Cell Sources

Multiple stem cell types have been investigated to differentiate into the tenogenic lineage and to promote tendon repair. When selecting an appropriate stem cell source, tendon stem/progenitor cells (TSPCs) and mesenchymal stem cells (MSCs) originating from several other tissues are suggested as suitable alternatives.

#### 2.2.1. Tendon Stem/Progenitor Cells

TSPCs first isolated from human and mouse tendon tissues were identified as multipotent cells by Bi et al. [18]. They exhibited the classical characteristics of adult MSCs, including expression of specific surface antigens, self-renewal ability, clonogenicity, and multilineage differentiation (adipogenic, osteogenic, and chondrogenic) capacity. Unlike other MSC types, TPSCs express high levels of tendon-related genes and tend to form tendon- and enthesis-like tissue after in vivo transplantation [18], indicating its high tendency toward tenogenic differentiation. Several studies have investigated the role of TSPCs in tenogenic differentiation and tendon repair [19–22]. Perucca Orfei et al. [19] studied in vitro TSPC cultivation at different cell densities. They indicated that TSPCs cultured at a high cell density exhibited a more elongated fibroblast-like morphology and a higher expression of tendon-related genes. Moreover, Durgam et al. [20] selected a horse as a clinically relevant animal model for investigating the effect of TSPC in vivo implantation on tendon healing. According to the results, autogenous TSPCs profoundly improved flexor tendon healing, with the repaired tendons exhibiting superior tensile stress and collagen alignment. Thus, TSPCs have been recognized as among the most ideal cell types for tendon repair.

However, TSPC application is associated with some limitations, such as the observed phenotypic drift during in vitro expansion [23]. In addition, because TSPCs comprise only a tiny fraction of cells in tendon tissue, a large volume of tissue is required for obtaining sufficient amounts of cells. Furthermore, donor site morbidity also restricts the clinical utilization of TSPCs [24].

#### 2.2.2. Bone Marrow-Derived Mesenchymal Stem Cells

Compared with TSPCs, bone marrow-derived MSCs (BMSCs) can be more easily obtained through iliac crest aspiration and massively expanded to a large amount in vitro [25]. BMSCs have a marked potential for tenogenic differentiation and tendon repair, as verified in a series of studies [26–28]. Wang et al. [26] revealed that bone morphogenetic protein (BMP)-14 strongly stimulated Scx and tenomodulin (Tnmd) expression in BMSCs, thus inducing a tenogenic phenotype in vitro. Renzi et al. [27] used BMSCs for in vivo tendon repair in an equine model. The injured animals recovered from their sports activities to a certain extent after the stem cell treatment.

However, whether BMSCs can maintain a stable tenogenic phenotype after implantation into the injury site remains elusive since ectopic ossification is frequently observed in many animal studies [10]. In addition, a concern exists that the biomechanical strength of the tendon repaired with BMSCs is not comparable with that of the native tendon [10]. Moreover, other difficulties, such as painful bone marrow-harvesting procedures and decreased stem cell quality in elderly donors, also restrain the wide application of BMSCs for tendon repair [29].

#### 2.2.3. Adipose-Derived Stem Cells

Adipose-derived stem cells (ASCs) are similar to BMSCs at morphological and molecular levels, and they are considered the most abundant and easily obtained MSCs. ASCs can be isolated from the subcutaneous adipose tissue through minimally invasive liposuction. Although ASCs are an alternative to BMSCs, a larger amount of bone marrow is required to produce the same amount of BMSCs [30]. This shows that ASCs are more accessible and less morbid during isolation. Using the connective tissue growth factor (CTGF) for promoting ASC tenogenic differentiation in vitro, Li et al. [31] demonstrated that CTGF dramatically increased Scx and Tnmd expression in mouse ASCs in a dose- and time-dependent manners. Similarly, Zarychta-Wiśniewska et al. [32] treated human ASCs with BMP-12 and induced tenogenic differentiation of these cells with elevated Scx expression. In another study, ASC-seeded scaffolds were transplanted into a 2 cm rabbit Achilles tendon defect. The cell-seeded constructs formed neotendon with a histological structure and tensile strength similar to those of native tendons [33].

Regarding its origin, ASCs exhibit an intrinsic tendency toward adipogenesis, and ectopic fat deposition was observed in animal experiments using ASCs for tendon repair [34]. More in-depth studies at the molecular level are warranted to uncover the exact mechanism underlying these phenomena.

#### 2.2.4. Embryonic Stem Cells and Induced Pluripotent Stem Cells

Embryonic stem cells (ESCs) are derived from the inner cell mass of blastocysts. They can differentiate into all cell lineages from the three germ layers: mesoderm, ectoderm, and endoderm [35]. Owing to their powerful differentiation capacity, they are considered valuable cell sources for regenerative medicine [36]. Barsby et al. [37] used transforming growth factor beta 3 (TGF-*β*3) to induce tenogenic differentiation of equine ESCs in a three-dimensional (3D) cultivation environment. They also exhibited strongly upregulated expression of tendon-related genes, including Tnmd, thrombospondin 4 (Thbs 4), tenascin C (Tnc), and collagen I (Col I). Moreover, in vivo studies have also shown great potential in promoting tendon healing. Watts et al. [38] reported that tissue repair can be improved based on histological and ultrasound outcomes in ESC-treated tendons. However, ESC application is associated with social and moral issues regarding embryonal tissue decomposition [35]. Moreover, the risk of tumor formation is another problem that cannot be overlooked [39].

The ethical and legal issues associated with the clinical application of ESCs can be overcome by using induced pluripotent stem cells (iPSCs). iPSCs are derived from differentiated somatic cells, and their potential in tenogenic differentiation has recently been investigated [40, 41]. Yang et al. [40] exposed equine tenocyte-derived iPSCs to cyclic uniaxial mechanical loading, which resulted in iPSC tenogenic differentiation along with increased Scx expression. This indicated that iPSCs can act as a promising cell source for tendon repair. However, challenges, such as a low-efficiency generation protocol and tumorigenesis after iPSC transplantation, remain unsolved, thereby limiting the clinical translation of the application of iPSCs for tenogenic differentiation [42].

## 3. Strategies for Tenogenic Differentiation

**Figure 2:**
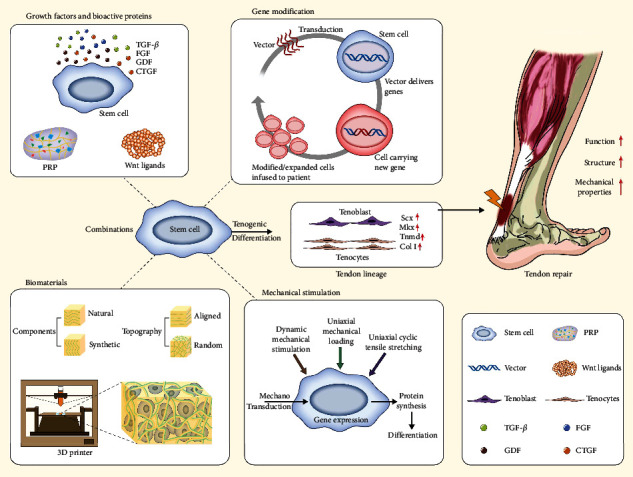
Conceptual framework summarizing the mostly reported strategies for tenogenic differentiation, including growth factors and bioactive proteins, gene modification, biomaterials, and mechanical stimulation.

The application of stem cells and their clinical translation are currently hampered because of the lack of a widely adopted protocol for effective and reproducible tenogenic differentiation. To improve the efficiency of a stem cell application for tendon repair, effective and reproducible strategies for tenogenic differentiation need to be urgently developed. Several tendon-specific biomarkers, such as Scx and Mkx, have been identified; however, more definitive biomarkers are required for understanding tendon differentiation cascades. Scx and Mkx are considered tendon-specific markers because they are continuously expressed during mature tendon formation [43]. In addition, early growth response proteins 1 (Egr1) and Egr2 are involved in tendon development. These proteins can also induce ectopic expression of tendon-specific markers [44]. Col I, Tnmd, and Tnc are tendon-related matrix proteins that are considered tenogenic markers for monitoring tendon development in the late embryonic development stage [45]. Thus, the aforementioned transcription factors and matrix proteins are usually considered molecular markers for determining whether tenogenic induction is successful. Four frequently reported strategies for inducing tenogenic differentiation in vitro and in vivo are discussed below (Figure 2).  Table 1 summarizes the application of growth factors and bioactive proteins, gene modification, biomaterials, and mechanical stimulation for tenogenic differentiation.

### 3.1. Growth Factors and Bioactive Proteins

Specific growth factors and bioactive proteins have a positive influence on the tenogenic differentiation of stem cells. In addition to the application of a single growth factor, various studies have applied a combination of multiple growth factors to enhance tenogenic differentiation.

#### 3.1.1. Transforming Growth Factor Beta

TGF-*β* is the most crucial signaling pathway activated during early tendon cell differentiation and tenogenesis [46]. Most tendons and ligaments in the limbs, trunk, tail, and head are lost when TGF-*β* signaling is disrupted in embryos [47]. TGF-*β* has been suggested to be a powerful inducer of tendon transcription factors [48–50]. It has a central role in promoting MSC tenogenesis [48]. All TGF-*β* isoforms have a remarkable effect on musculoskeletal tissues, but their influences on cell differentiation and biological behavior are different. Kuo et al. [6] first investigated different TGF-*β* isoforms in tendon development. They demonstrated the distinct spatiotemporal localization patterns of TGF-*β*1, -*β*2, and -*β*3 in the developing tendon, indicating that those isoforms have independent roles during tendon development. Moreover, they found that TGF-*β*2 and -*β*3 were most broadly expressed on day 14 of chick tendon development, whereas TGF-*β*1 expression was not detected on day 13 but became strong by day 16 [6]. Maeda et al. [51] revealed that TGF-*β*1 signaling is required for maintaining Scx expression in cultured tenocytes. TGF-*β*2 was found to be expressed in embryonic tendons, [6] and it ensured the tenogenic commitment of embryonic tendon cells [47]. TGF-*β*2 treatment induced a fibroblastic morphology in MSCs with increased Scx and Tnmd expression [49]. Moreover, TGF-*β* has been proven to be a critical player during tendon repair. In vivo treatment of injured tendons with TGF-*β* improved tendon repair, leading to tendons with a superior histological structure and biomechanical properties [52]. Interestingly, TGF-*β* exhibited spatiotemporal effects during tendon healing. Juneja et al. [53] reported a biphasic pattern of TGF-*β* expression during tendon healing, with a distinct TGF-*β*1 upregulation in the early healing phases, which gradually decreased thereafter. In the later phases, TGF-*β*3 expression increased and remained elevated. Chan et al. [54] also demonstrated temporal and spatial changes in the expression of TGF-*β* isoforms during tendon healing. They found that TGF-*β*1 expression was upregulated in the wound of the injured tendon at early tissue healing and was regressed to the lateral wound edges at later stages. TGF-*β*2 and -*β*3 showed a similar temporal change in expression; however, a timing difference in the application led to a difference in the expression of the three isoforms [54]. These studies have indicated that TGF-*β* isoforms have differential activities during tendon development and tissue healing. However, the underlying exact molecular mechanism needs deeper investigation.

Although TGF-*β* has a pivotal role in tendon healing, TGF-*β*1 often activates its downstream TGF-*β*1/Smad signaling pathway, which triggers profibrotic gene overexpression and leads to progressive fibrosis and scar formation [55, 56]. The two main downstream regulators, Smad2 and Smad3, promote TGF-*β*1-mediated tissue fibrosis. Conversely, Smad7 is a negative feedback regulator of the TGF-*β*1/Smad pathway that prevents TGF-*β*1-mediated fibrosis [55]. Administration of a neutralizing antibody to TGF-*β*1 in rabbit flexor tendons reduced scar and adhesion formation with an improvement of flexor tendon excursion, whereas simultaneous infiltration of a neutralizing antibody to TGF-*β*2 nullified this effect [57]. Further studies are required to design efficient methods for impeding the effects of the TGF-*β*1/Smad pathway in tissue fibrosis and scar formation.

#### 3.1.2. Connective Tissue Growth Factor

CTGF has been demonstrated to facilitate tendon regeneration through FAK/ERK1/2 signaling-mediated stimulation of endogenous TSPCs [58, 79, 80]. Li et al. [31] confirmed that treatment of mouse ASCs with CTGF promoted cell proliferation and stimulated the expression of tenogenic markers, including Scx and Tnmd. Furthermore, when ERK1/2 and FAK pathways were blocked, CTGF-induced tenogenic differentiation was suppressed. This suggested that CTGF induced ASC tenogenic differentiation through the aforementioned pathways.

CTGF also exhibited great potential for facilitating in vivo tendon repair. CD146 is among the specific markers expressed in TSPCs [18]. Lee et al. [80] demonstrated that CTGF delivery in vivo recruited endogenous CD146+ TSPCs for early tendon repair. In the later stage, CTGF promoted TPSC tenogenic differentiation with significantly elevated Col I, Tnmd, Scx, and Tnc expression. Furthermore, these effects improved tendon repair and led to better tendon structural reconstruction and functional restoration.

#### 3.1.3. Growth Differentiation Factor

The growth differentiation factor (GDF) is a protein subfamily belonging to the TGF-*β* superfamily. It is actively involved in the development of a wide range of tissues including the musculoskeletal system [61, 81]. GDF-8 was originally identified to prevent muscle growth and to be expressed in tendons [82]. According to Uemura et al. [83], GDF-8 promoted tenogenic differentiation of C2C12 myoblast cells with dramatic upregulation of tenogenic markers. When rat BMSCs were treated with 500 ng/mL GDF-8 [62], Col I, Scx, and Tnmd gene expression increased after 48 h of treatment and reached a peak at 72 h, and then, it gradually decreased from 96 h to 144 h. Ciardulli et al. [60] confirmed that GDF-5 dose-dependently promoted tenogenic differentiation of human MSCs. They treated the cells with 1, 10, and 100 ng/mL recombinant human GDF-5 (), and the highest concentration exerted the best effect on tenogenic induction. In an Achilles transection rat model, GDF-5, GDF-6, and GDF-7 expressions were detected during tendon healing, suggesting their involvement in tendon repair [84]. Interestingly, Würgler-Hauri et al. [85] reported that the expression of these GDFs was elevated in early tendon healing and decreased gradually over time in later stages, indicating their crucial role in early tissue healing.

Moreover, GDF-5 exhibited antifibrotic effects in tendon healing. Hasslund et al. [86] demonstrated that reconstructed flexor tendons treated with different dosages of GDF-5 exhibited comparable joint flexion function improvement. However, the lower GDF-5 doses were more potent in suppressing adhesions and fibrosis.

#### 3.1.4. Fibroblast Growth Factor

Fibroblast growth factors (FGFs) play an essential role in cell migration, proliferation, and differentiation [87]. FGF-2 is a potent mitogen for various cells, including MSCs and progenitor cells [88–90]. FGF-2 regulates MSC differentiation toward tendon lineage through the FGF/ERK/MAPK pathway [91]. Hankemeier et al. [92] reported that lower concentrations of FGF-2 (<10 ng/mL) were more beneficial for tenogenic differentiation than higher concentrations. Guo et al. [63] transfected human TSPCs with *FGF-2-*carrying lentivirus, and FGF-2 overexpression remarkably upregulated Col III and Scx expression. Tokunaga et al. [93] reported that FGF-2 administration induced Scx expression in TSPCs and promoted tendon healing 4 and 8 weeks after in vivo cell transplantation, indicating that FGF-2 is a positive regulator of tenogenic differentiation and tendon repair. However, Otsuka et al. [64] revealed that FGF-8b tended to reinforce chondrogenic differentiation while suppressing ASC tenogenic differentiation because the expression of tenogenic markers (Scx, Tnmd, and Tnc) and tendon extracellular matrix proteins (Col I and III) were downregulated. By contrast, the expression of the chondrogenic markers was upregulated after FGF-8b treatment. Thus, the effect of each FGF family member on tenogenic differentiation as well as the underlying molecular mechanisms need to be researched further.

#### 3.1.5. Combination of Different Growth Factors

Various growth factors are involved in tendon formation and development. The tendon repair process is complexly orchestrated by multiple growth factors and cytokines involved in different phases of the healing process and exhibiting diverse molecular effects [94]. Hence, applying a combination of growth factors seems more potent for promoting tenogenic differentiation and tendon repair than applying a single growth factor. Basically, numerous studies have investigated the application of a single growth factor for inducing stem cell differentiation toward tenogenic lineage in vitro, as well as for boosting the tissue healing process in vivo. Simultaneous or sequential exposure of stem cells to combinations of multiple growth factors, designed to integrate and amplify the tenogenic differentiation effects, has been investigated frequently. However, consensus on the most potent combination of growth factors for tenogenic differentiation is lacking.

In both 2D monolayer and 3D hydrogel cultivations, a combination of BMP-14, VEGF, and TGF-*β*3 enhanced tenogenic differentiation of rabbit BMSCs with higher Col Ia1, Col IIIa1, Tnc, and Tnmd expression compared with an individual growth factor and other tested combinations [95]. Additionally, Perucca Orfei et al. [65] highlighted the significance of differential timing of application of growth factors in tenogenic differentiation. Their studies have revealed that TGF-*β*3 was the main inducer of the tendon-specific marker Scx in early tenogenic differentiation, whereas it inhibited the tendon marker, decorin, expressed during late tenogenic differentiation. Moreover, other tested growth factors, such as BMP-12, FGF, and ascorbic acid, dominantly induced decorin expression in the later stage. This suggests the advantages of a stepwise protocol using different growth factors at different time courses for tenogenic differentiation with a properly organized extracellular matrix. Similarly, Yin et al. [96] reported that TGF-*β*1 was the main inducer of the tendon-specific transcription factor, Scx, and CTGF induced the expression of the matrix protein, Tnmd. Accordingly, they developed a stepwise protocol wherein TGF-*β*1 stimulation followed by a combination with CTGF for another 7 days led to an efficient BMSC tenogenic differentiation. Furthermore, an injured patellar tendon treated with tenogenic-induced BMSCs exhibited better in vivo tendon repair with superior structural and biomechanical properties than the control group. These studies have indicated that the combination of different growth factors and the timing of their application need to be elaborately considered in the future to maximize their effects on tenogenic induction and tendon healing.

In summary, the combined application of growth factors is crucial for tenogenic differentiation, as well as for achieving better tendon healing. However, more studies are warranted to clarify the optimal timing, appropriate combination, interaction, and dosage of growth factors in tenogenic differentiation and tendon repair.

#### 3.1.6. Wnt Ligands

The Wnt signaling pathway is fundamentally important for limb development and plays a vital role in tendon/ligament formation during embryogenesis [46]. Wnt/*β*-catenin signaling activation in BMSCs induced their tenogenic differentiation with upregulated expression of Tnmd and other tendon-related matrix genes, decorin, and fibromodulin (Fmod) [66]. However, the expression of the two most crucial tendon-specific transcription factors, Scx and Mkx, was not affected. This indicated that the role of Wnt/*β*-catenin signaling in tenogenic differentiation was independent of Scx and Mkx. Furthermore, Wnt4 and Wnt5a mediated mechanical stimulation-mediated tenogenic differentiation of human MSCs [97]. In the future, conducting studies testing the effects of ectopic Wnt application for tenogenic differentiation and tendon repair would be meaningful.

#### 3.1.7. Platelet-Rich Plasma

Platelet-rich plasma (PRP) is concentrated with various whole blood-derived growth factors. PRP stimulated migration, proliferation, and tenogenic differentiation of paratenon-derived cells in vitro, as indicated by significantly elevated expression of tendon-related markers [67]. Moreover, combining PRP with other bioactive factors, it would be promising for enhancing tenogenic differentiation and tendon healing. However, the exact components that play a central role during certain biological processes and the underlying molecular mechanisms remain largely unknown, which has impeded the wide application of PRP.

### 3.2. Gene Modification

Gene modification is another strategy frequently used for improving tenogenic differentiation. Several studies have investigated the role of gene modifications in controlling tenogenic differentiation, including gene delivery growth factors, transcription factors, and noncoding RNA.

Growth factors such as BMP-12 and CTGF are crucial regulators of tenogenic differentiation. Xu et al. [68] transfected TSPCs with BMP-12 and CTGF through recombinant adenovirus infection, and then, these cells were transplanted into rat patellar tendon window defect for tendon repair in vitro. The expression of tendon-related genes, Col I, Col III, Tnc, and Scx, was upregulated in TSPCs. Meanwhile, the expression of the osteogenic marker Runx 2, adipogenic marker PPAR, and chondrogenic marker Sox9 was downregulated. Moreover, BMP-12- and CTGF-overexpressing TSPCs strongly promoted tendon healing in vivo. Guo et al. [63] transfected human TSPCs with *FGF-2-*carrying lentivirus, and FGF-2 overexpression remarkably enhanced tenogenic induction in vitro, as well as improved tendon repair in vivo.

Transcriptional factors Scx, Mkx, and Egr play critical roles in tendon development and differentiation. Alberton et al. [69] reported that ectopically expressed Scx in BMSCs (BMSC-Scx) effectively directed their commitment into tendon progenitors based on the results of remarkably upregulated Tnmd, Col I, and several other tendon-related proteoglycans, as well as the cells failed to differentiate into chondrogenic and osteogenic lineages. Otabe et al. [70] transfected human BMSCs with the Mkx adenoviral vector, and the expression of tendon-related genes Col I, Tnmd, and Tnc was significantly elevated after 1 week. The transcriptional factor Egr was found to be involved in tendon differentiation by regulating tendon-related collagen production [98]. Ectopic Egr1 expression in TSPCs through Egr1-expressing plasmid transfection induced tenogenic differentiation of these stem cells. In addition, Egr-1-suppressed PPAR*γ*, Runx2, and Sox9 expression was suppressed [71]. Moreover, Egr1-overexpressing TSPCs promoted in vivo rotator cuff repair in a rabbit model through the BMP12/Smad1/5/8 signaling pathway [71].

Noncoding RNA, such as microRNA (miRNA), is involved in tendon injury healing [30]. By directly binding to the 3′-untranslated target mRNA region, miRNA negatively regulates the target gene expression by preventing transcript translation. The expression of ROCK1, which has been demonstrated to be increased in aged or senescent TSPCs [99], was primarily suppressed by miR-135a overexpression in young TSPCs based on Chen et al.'s study [100]. They confirmed that miR-135a overexpression in TSPCs suppressed senescence and enhanced their tenogenic differentiation [100]. On the other hand, in miR-378a knock-in transgenic mice, Liu et al. [72] found that miR-378a suppressed tenogenic differentiation and tendon repair by inhibiting collagen and ECM production both in vitro and in vivo.

Furthermore, emerging evidence has shown that long noncoding RNA (lncRNA) significantly affected cell differentiation and tissue regeneration [101, 102]. Lu et al. [73] discovered that stable lncRNA H19 overexpression in human TSPCs improved tenogenic differentiation and promoted in vivo tendon repair in a mouse model by targeting miR-29b-3p and activating TGF-*β*1 signaling. In summary, noncoding RNAs have gradually become a research hotspot. However, further studies are required to thoroughly understand the exact molecular mechanism underlying its role in tenogenic differentiation and tendon healing, which will also facilitate the identification of tendon-specific biomarkers.

### 3.3. Biomaterials

A microenvironment containing macromolecular components with unique biophysical, biochemical, and biomechanical attributes is the key in governing cell functions, such as migration, proliferation, and differentiation [103]. Natural or synthetic biomaterials provide a 3D environment that supports cell proliferation and matrix remodeling and have been extensively investigated for directing stem cell fate and molecular behaviors [104]. Biomaterial properties, such as stiffness, act as critical factors for regulating cell function and differentiation. A newly developed scaffold which mimics the stiffness of the young brain is molecularly and functionally rejuvenated aged oligodendrocyte progenitor cells [105]. Similarly, Yin et al. [104] reported a nanofibrous RADA-based hydrogel that mimics the ultrastructure and mechanical property of natural tendon ECM-rejuvenated aged human TSPCs to a phenotype resembling that of young donor cells with upregulated tenogenesis-related genes. This indicated that the structural and mechanical properties of the surrounding matrix could deeply affect cell behavior and differentiation.

Decellularized tendons tend to reserve specific components of native tendon ECM as well as various bioactive factors. By seeding human ASCs in a tendon ECM-supplemented scaffold, Yang et al. [74] demonstrated that the scaffold promoted the proliferation and tenogenic differentiation of ASCs. Moreover, the expression of osteogenic markers, including Runx 2, Alp, and Ocn, was suppressed, suggesting that the tendon-derived decellularized matrix specifically supported tenogenic differentiation but prevented differentiation into the osteogenic lineage. Ning et al. [106] found that a decellularized tendon hydrogel developed from *Macaca mulatta* Achilles tendons retained stromal cell-derived factor-1 and Fmod inherent to the native tendon matrix microenvironment. When *M. mulatta* TSPCs were cultured on the hydrogel, Scx, Tnmd, and Tnc expression of the cells was significantly upregulated. Moreover, combining the tendon-derived matrix with growth factors has been investigated for promoting tenogenic differentiation. Yang et al. [107] reported that a decellularized tendon ECM augmented TGF-*β*3-mediated tenogenic differentiation of human ASCs along with elevated Scx expression.

The topographical structure is another critical factor directing stem cell fate and differentiation. A mature native tendon matrix presents a hierarchical structure with highly parallelly aligned collagen fibers. Artificial microenvironments mimicking the topographical structure of native tendons have been created to facilitate stem cell differentiation toward the tenogenic lineage [108, 109]. Biomaterials with parallelly aligned topography are advantageous in inducing tenogenic differentiation [110, 111]. Zhang et al. [111] showed that the electrospun scaffold of aligned poly (l-lactic acid) (PLLA) fibers resembling the tendon ultrastructure promoted TPSC tenogenic differentiation in vitro and boosted in vivo Achilles tendon healing with superior structural and mechanical properties. Xu et al. [112] reported that Col I hydrogels with aligned iron oxide nanoparticles (IOP) induced human TSPC growth in a manner along with the aligned IOP rows. Moreover, this anistropic construct promoted TPSC tenogenic differentiation and significant upregulation of tendon gene markers. The collagen fibril diameter in the tendon decreases upon injury or with aging, and thus, controlling the fiber diameter is also critical while designing scaffolds [75]. Erisken et al. [75] demonstrated that scaffolds with nanofibers induced a higher number of cells, total collagen, and proteoglycan production, while those with microfibers stimulated the expression of tenogenic markers, collagen I, III, and V, and Tnmd of tendon fibroblasts. Cell morphology is profoundly associated with the determination of MSC differentiation. Elongated cell morphology is essential for maintaining a tenogenic phenotype [113]. Shi et al. [114] studied TSPC differentiation directed by a specifically designed topographic surface and found elongated TSPCs induced by the parallel microgrooved polydimethylsiloxane membrane exhibited improved expression of tenogenic marker genes. In addition, the differentiation of elongated TSPCs toward chondrogenic and adipogenic lineages on this topography was suppressed.

To summarize, fine-designed biomaterials with proper components, a topographical structure, and biomechanical properties exhibit a powerful potential in directing the tenogenic differentiation of stem cells. These biomaterials will be a major future research direction for facilitating tendon healing.

### 3.4. Mechanical Stimulation

Mechanical stimulation is significantly involved in all stages of the musculoskeletal system lifecycle [12]. Mechanotransduction can be translated into signaling cascades, which ultimately regulate gene expression and direct cell differentiation [115]. In fact, the tendon is a mechanosensitive tissue and is constantly subjected to uniaxial mechanical stretch parallel to cell orientation; hence, tendon cells maintain an innate morphology and tenogenic phenotype [116]. In addition, dynamic mechanical stimulation is required for mature tendon development along with the advanced form of tendon matrix proteins (Col I and III) and stable expression of tendon-specific transcription factors, such as Scx [117]. Compared with the control group without mechanical stimulation, dynamic stretch stimulated collagen matrix deposition and upregulation of tenogenic marker genes in human ASCs seeded on the woven nanofibrous polycaprolactone scaffold [76]. Wang et al. [77] reported that uniaxial mechanical loading induced tenogenic differentiation and neotendon formation in both mouse and human TSPCs in 3D constructs.

However, different stimulation regimes and various parameters, such as strain amplitude, loading frequency, and intensity, have differential effects on cell differentiation. Wang et al. [77] demonstrated that uniaxial loading induced tenogenic differentiation and neotendon formation of mouse and human TSPCs in 3D constructs, while biaxial loading induced osteogenic, adipogenic, and chondrogenic differentiation of TSPCs. This indicated the importance of appropriate mechanobiological stimulation for tenogenic induction. Uniaxial cyclic tensile stretching at 8% strain led to exclusive BMSC tenogenic differentiation and comparable protein and gene expression to primary tenocytes and inhibited nontenogenic lineage differentiation [118]. Xu et al. [78] reported that cyclic tensile strain at different frequencies (0.3, 0.5, and 1.0 Hz) with the same amplitude exerted different effects on TPSC proliferation and Col I, tenascin-C, Tnmd, and Scx expression. The most prominent tenogenic induction was achieved at 0.5 Hz frequency. Subramanian et al. [119] studied the optimal parameters of uniaxial loading for tenogenic differentiation of MSCs. They found that mechanical loading of 2% strain and 0.1 Hz frequency induced tenogenic differentiation of ASCs encapsulated within a collagen scaffold. Moreover, with this loading regime, no potential for cross-differentiation to osteogenic, chondrogenic, and myogenic lineages was observed. In addition, loading intensity is believed to influence cell differentiation. Moderate intensity tends to promote tendon-related gene expression and neotendon formation, whereas intensive intensity usually leads to osteogenic differentiation [120]. In the future work, the effects of appropriate mechanical stimulation regimes with optimal parameters on tenogenic differentiation need to be investigated in-depth to establish reliable standards for effective and specific tenogenic differentiation.

## 4. Conclusion and Future Perspectives

In recent years, a series of promising treatments including stem cell-based therapies for tendon repair have achieved a certain degree in experimental research. However, a considerable gap continues to exist between the basic findings and clinical applications. The main reasons are that the exact mechanisms underlying tenogenic differentiation and tendon healing remain largely unclear. Moreover, no widely adopted protocol has been established for effective and reproducible tenogenic differentiation. This work reviews stem cell applications for tendon repair and highlights the unique advantages of reported strategies for tenogenic differentiation, including growth factor application, gene modification, biomaterial application, and mechanical stimulation. Current experimental data support these strategies for triggering tenogenic differentiation. Tendon is a mechanosensitive tissue, and tendon development and tissue healing are complexly orchestrated by multiple factors with diverse molecular effects. Hence, investigating the use of a combination of the aforementioned factors warrants further explorations, so that a powerful and specific tenogenic differentiation protocol and advanced tendon repair strategy can be developed.

However, a series of points remains to be noted: (1) the tenogenic phenotype needs to be more clearly defined; (2) universal protocols for promoting tenogenic differentiation need to be established; (3) consensuses need to be reached for the parameters for each strategy, such as the optimal timing and dosage of growth factors, as well as the appropriate combination of different factors to be considered for improving the efficiency of tenogenic differentiation and avoid differentiation toward the nontenogenic lineage; and (4) attention has to be paid to the safety and moral issues of stem cell application in clinical applications [121]. Future studies are encouraged to focus on solving the aforementioned problems stated and speed up the transfer of stem cell-based regenerative medicine efforts from bench to bedside. According to our perspective, the use of a combination of growth factors at a suitable dosage and time, biomaterial application, and mechanical stimulation according to appropriate parameters can induce optimal tenogenic differentiation of stem cells and achieve advanced tendon healing.

## Figures and Tables

**Table 1:** Strategies in promoting tenogenic differentiation.

Treatment	Study type	Animal model	Cell source	Outcomes	References
*Growth factors and bioactive proteins*					
TGF-*β*1	In vivo	Rat	—	Biomechanical strength↑	Arimura et al. Am J Sports Med [59]
TGF-*β*2	In vitro	—	MSCs	Scx and Tnmd↑, N-cadherin and cadherin-11↓	Theodossiou et al. Biochem Biophys Res Commun. [49]
TGF-*β*3	In vivo	Rat	—	Histological and biomechanical levels↑	Han et al. Arthroscopy. [52]
CTGF	In vitro and in vivo	Rat	TSPCs	Col I, Tnmd, Scx, and Tnc↑ in vitro and promoting tendon healing with well-aligned collagen fibers in vivo	Lee et al. J Clin Invest [58]
GDF-5	In vitro	—	BMSCs	Col I, Col III, Dcn, Scx, Tnc, and Tnmd↑	Ciardulli et al. Int J Mol Sci [60]
GDF-6	In vitro and in vivo	Rat	BMSCs	Scx and Tnmd↑ in vitro and showing neotendon formation in vitro	Chai et al. Chin Med J (Engl). [61]
GDF-6 and 7	In vitro	—	ESCs	Tnc, Col I, and III↑	Dale et al. Tissue Eng Part A. [36]
GDF-8	In vitro	—	BMSCs	Col 1A, Scx, and Tnmd ↑	Le et al. J Hand Surg Asian Pac Vol [62]
FGF-2	In vitro and in vivo	Rat	TSPCs	Scx and Col III↑ in vitro and enhancing tendon healing in vivo	Guo et al. Biochem Biophys Res Commun [63]
FGF-8b	In vitro	—	ASCs	Scx, Tnmd, and Tnc↓, Col I and III↓	Otsuka et al. Stem Cell Res [64]
TGF-*β*3 + BMP-12+ IGF1 + CTGF+FGF + AA	In vitro	—	MSCs	Scx↑ (induced by TGF-*β*3) and decorin↑ (induced by BMP-12, FGF and AA)	Perucca Orfei et al. Int J Mol Sci [65]
Wnt ligands	In vitro	—	BMSCs	Tnmd, decorin, and Fmod↑	Miyabara et al. J Equine Sci. [66]
PRP	In vitro	—	Paratenon-derived cells	Scx and col I↑	Imai et al. Sports Health [67]
*Gene modification*					
Transfection with BMP-12 and CTGF	In vitro and in vivo	Rat	TSPCs	Tnc, Col I and III↑ in vitro and presenting better outcome on histology level in vivo	Xu et al. J Biomech [68]
Transfection with Scx	In vitro	—	BMSCs	Tnmd, Col I and several tendon-related matrix proteoglycans↑	Alberton et al. Stem Cells Dev [69]
Transfection with Mkx	In vitro	—	BMSCs	Tnmd, Tnc and Col I↑	Otabe et al. J Orthop Res [70]
Transfection with Egr1	In vitro and in vivo	Rabbit	TSPC	Scx and Tnmd↑ in vitro and promoting rotator cuff repair in vivo	Tao et al. Cell Physiol Biochem [71]
MiR-378a	In vitro and in vivo	Mice	TSPCs	Scx, Mkx, Fmod, Thbs4, Col I and III↓ in vitro, and Thbs4, Col I↓ in vivo	Liu et al. Stem Cell Res Ther [72]
lncRNA H19	In vitro and in vivo	Mice	TSPCs	Sxc, Mkx, Tnmd, Fmod, Dcn, and Col I↑ and enhancing tendon healing in vivo	Lu et al. FASEB J [73]
*Biomaterials*					
Tendon ECM-supplemented scaffold	In vitro	—	ASCs	Scx, Tnmd, and Tnc↑	Yang et al. Biomaterials [74]
Nanofiber and microfiber scaffolds	In vitro	—	Tendon fibroblasts	Cell number, total collagen and proteoglycan production↑ in nanofibers; Tnmd, Col I, III, and V↑ in microfibers	Erisken et al. Tissue Eng Part A [75]
*Mechanical stimulation*					
Dynamic stretch+ woven nanofibrous PCL scaffold	In vitro	—	ASCs, tenocytes, and HUVEC	Scx, Tnmd, Tnc, VEGFA, and Col I and III↑, as well as the total collagen content↑	Wu et al. Acta Biomater [76]
Uniaxial loading on 3D constructs	In vitro	—	TSPCs	Scx, Mkx, Tnmd, Col I I, and neotendon formation	Wang et al. FASEB journal [77]
Cyclic tensile strain	In vitro	—	TSPCs	The expression of Col I, TNc, Tnmd, and Scx was most apparant at 0.5 Hz frequency with the same amplitude and at 4% amplitude with the same frequency	Xu et al. Biomed Res Int [78]

Abbreviations: TSPCs: tendon stem/progenitor cells; BMSCs: bone marrow stromal cells; ASCs: adipose-derived mesenchymal stem cells; ESCs: embryonic stem cells; MSCs: mesenchymal stem cell; HUVEC: human umbilical vein endothelial cells.
